# Modern contraceptive utilization and associated factors among street working reproductive age women in Ethiopia: A systematic review and meta-analysis

**DOI:** 10.1371/journal.pone.0312569

**Published:** 2024-12-27

**Authors:** Bekalu Getnet Kassa, Basazinew Chekol Demilew, Fentahun Yenealem Beyene, Habtamu Gebrehana Belay

**Affiliations:** 1 Department of Midwifery, College of Health Sciences, Debre Tabor University, Debre Tabor, Ethiopia; 2 College of Nursing and Health Science, Flinders University, Adelaide, Australia; 3 Department of Anesthesia, College of Health Sciences, Debre Tabor University, Debre Tabor, Ethiopia; 4 Department of Midwifery, College Medicine and Health Sciences, Bahir Dar University, Bahir Dar, Ethiopia; University of Foggia: Universita degli Studi di Foggia, ITALY

## Abstract

**Background:**

Street women are women, who make their living on the streets by begging, sleeping in the streets, or on the sides of roads. They are the most marginalized and neglected segment of society, with little access to health care, including modern contraception, and a lack of knowledge about health services, particularly in Ethiopia. Therefore, this study aimed to examine modern contraceptive utilization and associated factors among street women.

**Methods:**

A systematic review and meta-analysis were conducted on observational community-based studies published from January 2010 up to February 1, 2023. PubMed, Google Scholar, HINAR, Scopus, Web of Sciences, and grey literature were used to search primary studies. We used Microsoft Excel for data entry and extracting data. STATA-17 statistical software was used to analyze the data as well and I^2^ tests evaluated study heterogeneity. The pooled utilization of the modern contraceptive method was predicted using a random-effect model.

**Results:**

A total of eight studies were included. The pooled prevalence of modern contraceptive utilization was 51.89% (95% CI: 40.89–62.9). Being married (OR = 4.22, 95%CI, 2.75–6.49); facing a history of sexual assault/rape at a street life (OR = 3.59, 95%CI, 2.46–5.23); age between 25–35 years (OR = 3.27, 95%CI, 1.73–6.18), getting advise from the health professionals (OR = 6.23, 95%CI, 1.44–27.07), having a history of pregnancy in street life (OR = 1.90, 95%CI, 1.28–2.81) and no more children wanted (OR = 2.47, 95%CI, 1.52–5.00) were significantly associated with modern contraceptive utilization.

**Conclusions:**

The pooled modern contraceptive utilization was suboptimal. Every concerned body or stakeholder should give more consideration to their lifestyles and living conditions by raising awareness and providing access to contraception.

## Introduction

Family planning is defined as the ability of individuals and couples to anticipate and attain their desired number of children and the spacing and timing of their births through the use of contraceptive methods [1, 2]. These contraceptive methods include; pill, injectable, male and female condoms, emergency contraception, implants, intrauterine contraceptive device, female and male sterilization, standard day method, and lactational amenorrhea method [3–5].

Street women are women who make their living on the streets by begging, sleeping in the streets, or on the sides of roads [4]. They are the most marginalized and neglected segment of society, with little access to health care, including modern contraception, and a lack of knowledge about health services [6].

Every day, street women are exposed to situations that make them vulnerable to sexual and reproductive health issues especially a high-risk group for unintended pregnancies, sexually transmitted infections, and HIV/AIDS [7, 8]. According to research, women who live on the streets are less likely to benefit from basic reproductive health services, such as modern contraceptives [9]. This contraceptive utilization is influenced by a variety of factors, including socioeconomic status, knowledge about contraceptives, attitudes about contraceptive issues, residential area, educational status, contraceptive counseling received contraceptive provider attitudes, and cultural values, norms, and beliefs [10–12].

In Ethiopia, there is no comprehensive report regarding the use of modern contraceptive methods; but some primary studies have been conducted in different areas to examine modern contraceptive utilization and associated factors among street women. These studies had inconsistent results across each study. Hence, this systematic review and meta-analysis aimed to synthesize the pooled utilization of modern contraceptives and its associated factors among street women in Ethiopia. Thus, this study will provide input information for policymakers, and concerned shareholders for designing strategies to increase the use of contraceptives among those street women. Furthermore, the evidence bred from this review can be used as input for researchers who intend to make further investigations in this area.

## Methods and materials

### Study design

Systematic review and meta-analysis were conducted, and the study was carried out based on the Preferred Reporting Items for Systematic Reviews and Meta-Analyses (PRISMA) checklist [13] (**S1 Table**).

### Eligibility criteria

**Inclusion criteria.** Articles reporting the utilization of modern contraceptive methods and associated factors among street working women in Ethiopia were included in this study. In particular, studies that were included in this study considered the following criteria;

**Study area:** Only studies were conducted in Ethiopia.

**Study design:** All observational studies (cross-sectional, case controls, and cohort).

**Language**: The articles were published only in the English language.

**Population:** Studies that were conducted among street-working reproductive-age women.

**Publication condition:** Both published and unpublished articles.

**Publication year:** All publications reported from January 2010 up to February 01, 2023.

**Outcome:** Utilization of modern contraceptive methods among street working reproductive-age women.

### Operational definitions

**Street women:** Women who make their living on the street by begging, and sleeping on the street or roadsides which include both off-street women and on-street women

**On-street women:** women who had no formal home (homeless) who live and sleep on streets, verandas, and balconies

**Off-street women:** women who have houses to go to sleep at night while making their living on the street or begging for money on the street and returning to their formal homes at night to sleep

### Eligibility criteria

The review covered research done in Ethiopia between 2010 and February 2023 with full-text articles written in English. We excluded articles that did not specify the research population, qualitative studies, review articles, case reports, case studies, basic descriptive studies without regression analysis, and duplicate publications.

### Searching strategy and data sources

Published articles assessing modern contraceptive utilization and its associated factors in Ethiopia were included and a systematic search through electronic database systems including PubMed, Google Scholar, HINAR, Scopus, Web of Sciences, and Grey literature was used. We also conducted direct hand searches on Google and cross-reference lists were used. The PECO (Population, Exposure, Comparison, and Outcomes) search format has used this review to search for pertinent studies.

The search was performed by three authors using comprehensive searching strategies. Initially, articles were searched by examining the full titles; and then by using keywords (Modern[All Fields] AND ("contraceptive agents"[All Fields] OR "contraceptive devices"[MeSH Terms] OR ("contraceptive"[All Fields] AND "devices"[All Fields]) OR "contraceptive devices"[All Fields] OR "contraceptive"[All Fields] OR "contraceptive agents"[MeSH Terms] OR ("contraceptive"[All Fields] AND "agents"[All Fields]) OR "contraceptive agents"[All Fields]) AND ("statistics and numerical data"[Subheading] OR ("statistics"[All Fields] AND "numerical"[All Fields] AND "data"[All Fields]) OR "statistics and numerical data"[All Fields] OR "utilization"[All Fields]) AND associated[All Fields] AND factors[All Fields] AND street[All Fields] AND ("women"[MeSH Terms] OR "women"[All Fields]) AND ("Ethiopia"[MeSH Terms] OR "Ethiopia"[All Fields]). In addition, to retrieve relevant unpublished studies, Ethiopian universities’ digital libraries were used (**S2 Table**).

### Identification and study selection

All selected studies were exported to the Endnote X7 reference manager software. Studies were screened after reading the title and abstracts. Three authors screened and assessed articles separately. The full text of the study was further assessed based on aims, methodology, participants, and their findings of the study. Any disagreements were resolved through discussion and consensus.

### Quality assessment

The quality of included articles was assessed by the Newcastle-Ottawa scale quality assessment tool for cross-sectional studies quality assessments [14]. The qualities of each study were weighted by three authors independently using Newcastle-Ottawa scale quality assessment tool criteria. Those primary studies with a medium score (satisfying 50% quality evaluation criteria) and high quality ((≥7 out of 10) were included in this study. The three investigators’ differences were managed by taking the average score of their quality evaluation outcomes (**S3 Table**).

### Data abstraction

Data were extracted by using a standardized data extraction format. The format includes the primary author, year of publication, study setting, sample size, study design, response rate, prevalence, and associated factor with a 95% confidence interval.

**The outcome of interest.** The primary outcome variable was modern contraceptive utilization. This study also assessed the association between selected independent variables with modern contraceptive utilization. The independent variables included in this study desired to have additional children, history of pregnancy at street life, being married, being educated, faced a history of sexual assault/rape, women who have children after joining a street life, getting advice from a health professional, and age between 25–34 years.

### Publication bias and heterogeneity

Electronic database searches were used to minimize the risk of bias. The authors’ cooperative work was also critical in reducing bias, selecting articles based on clear objectives and eligibility criteria, deciding the quality of the studies, and extracting and compiling the data. We also examine publication bias with a visual inspection of the funnel plot [15]. Also, Egger’s correlation tests at a 5% significant level were conducted to assess the presence of publication bias [16]. Furthermore, to reduce the random variations among the primary study’s point estimates, subgroup analysis was conducted by study regions and sample size. Sensitivity analysis was also done to identify the potential source of heterogeneity. Heterogeneity across studies was evaluated using inverse variance (I^2^) statistics with its corresponding p-value using the random-effect model.

### Statistical analysis

We used Microsoft Excel for data entry and Stata version 17 software for analysis. The associated factors of modern contraceptive utilization were examined based on eligibility criteria. We considered at least two studies that reported one common associated factor of modern contraceptive utilization with their measure of effect and 95% confidence interval (CI). A random effects model based on the DerSimonian-Laird method was considered to assess for variations between the studies. The results were presented using texts, tables, and forest plots with measures of effect and a 95% confidence interval. Statistical heterogeneity was tested via the I^2^ statistics at a p-value of ≤ 0.05 [17].

### Ethics approval and consent to participate

This is not applicable because the study is a review, and the data used were obtained from already published materials.

## Results

**Fig 1:**
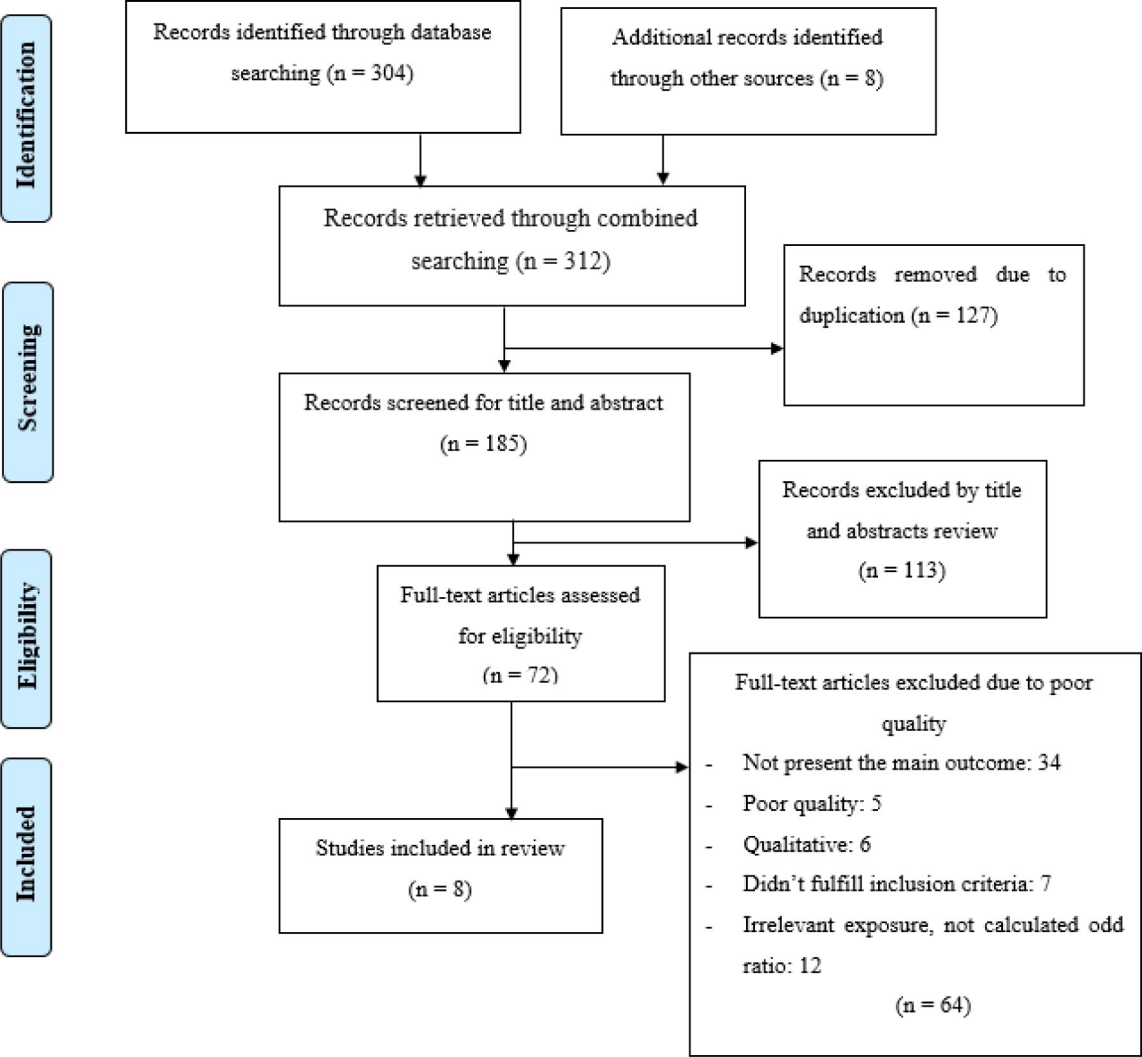
PRISMA flowchart diagram of the study selection for systematic review and meta-analysis on modern contraceptive utilization among street women in Ethiopia.

A total of 312 studies were retrieved by electronic search. Of these, 127 articles were removed due to duplication, and 185 articles were reviewed further for inclusion. Out of this article, 113 studies were excluded due to irrelevance, and 64 were excluded due to limited statistical analysis, an irrelevant target population, and an inconsistent study report. Finally, the study comprised eight articles that met the inclusion criteria and were included in this systematic review and meta-analysis (**Fig 1**).

This systematic review and meta-analysis included eight qualified observational studies reported in English from three regions and two city administrations. A total of 2504 street women participated in this study. The sample size ranged from 84 in the Addis Ababa city administration [18] to 641 in the Amhara region [19]. Modern contraceptive utilization was reported between 37.4% [20] and 78% [18] (**Table 1**). Concerning the quality score of the primary studies, three studies had a quality score of eight, two studies had a quality score of seven, and the remaining three had a quality score of nine. Hence, all of them had a good and above-quality score (**S2 Table**).

**Table 1:** Summary of eight observational studies included in this study assessing modern contraceptive utilization and associated factors among street women in Ethiopia, 2023.

S.N	Authors	Year	Regions	Study design	Study setting	Sample size	Population	Outcome	Prevalence	Factors
1	Kettema et al. [21]	2020	Amhara	Cross-sectional	Community based	641	Reproductive age street women	Modern contraceptive utilization	38.9	Having ≥ 3 children, desiring to have additional children, history of pregnancy, no contraceptive mentioned.
2	Engdaw A. [22]	2016	Amhara	Cross-sectional	Community based	238	Reproductive age street beggar	Modern contraceptive use	48.9	Married, educated, history of sexual assault/rape, getting advice from a health professional, living in a rented house.
3	Guta et al. [4]	2021	Dire Dawa	Cross-sectional	Community based	615	Reproductive age street women	Modern contraceptive utilization	50.3	Age 25–34 years, distance from a nearby health facility within 30 minutes, getting advice from health professionals, discussing with their partner about the use of modern contraceptives, history of pregnancy, women who faced rape in a street, desire to have a child.
4	Gebremeskel et al. [9]	2019	Oromia	Cross-sectional	Community based	163	Reproductive age street women	Modern contraceptive utilization experiences	43.2	Education, age, no of children
5	Alemu et al. [20]	2019	SNNPR	Cross-sectional	Community based	381	Reproductive age street beggar	Modern contraceptive utilization	37.4	Religion, husband-approved utilization, women who have children after joining a street life
6	Megabiaw B. [23]	2021	Amhara	Cross-sectional	Community based	204	Reproductive age street	Modern contraceptive use	47.1	Age 25–34 years, married, family size> 4, sexual assault at a street life
7	Habtemariam K. [24]	2014	Addis Ababa	Cross-sectional	Community based	84	Reproductive age street girls	Knowledge, attitude and practice of modern contraceptive use	78	Age 20–24 years, education
8	Alemayehu B. [25]	2018	Addis Ababa	Cross-sectional	Community based	178	Reproductive age street	Modern contraceptive utilization	71.3	Perceived treatment, lack of knowledge, beliefs

### Modern contraceptive utilization

**Fig 2:**
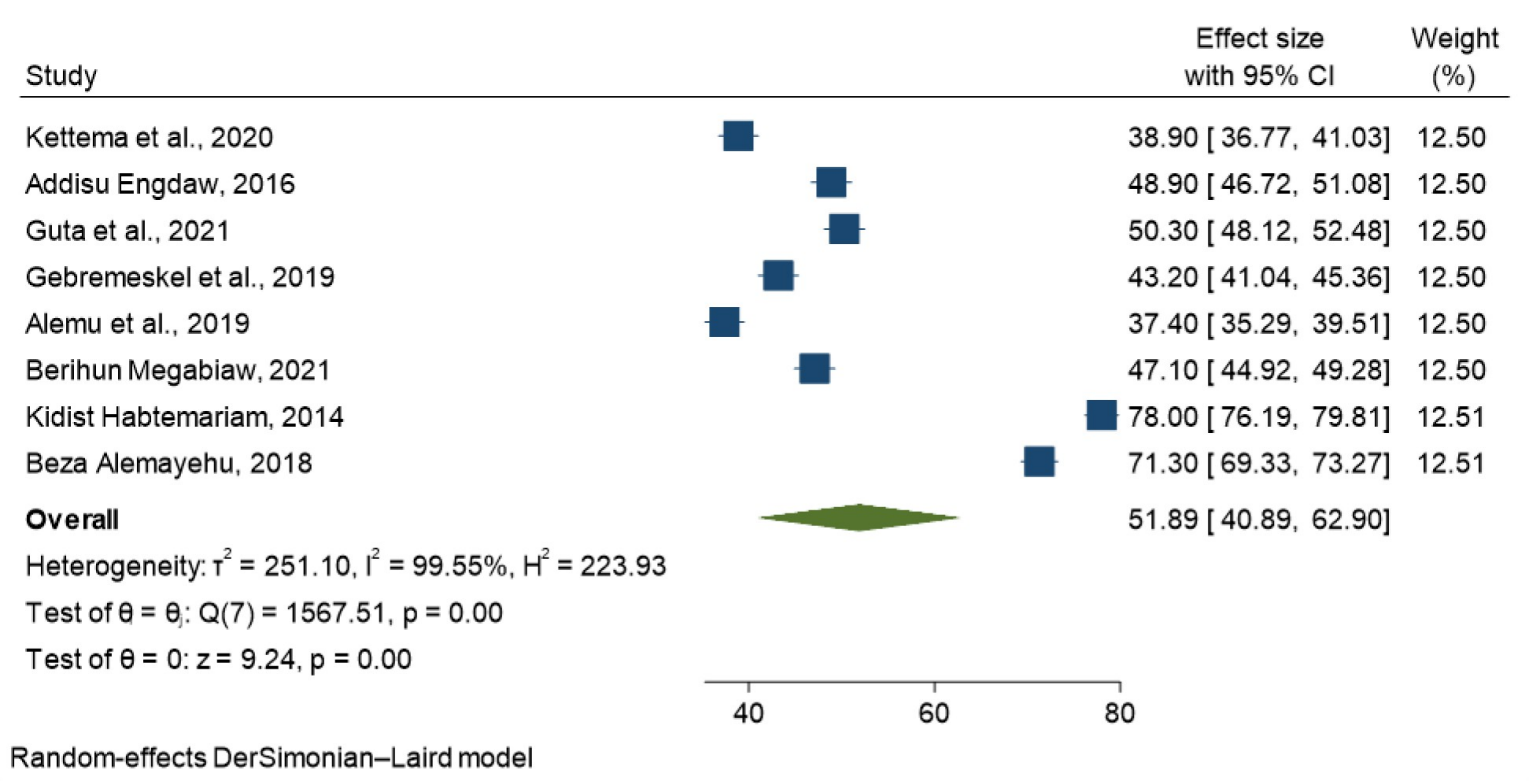
Forest plot of the pooled modern contraceptive utilization in Ethiopia, 2023.

Based on the random effect model, the overall pooled modern contraceptive utilization among street reproductive-age women in Ethiopia was 51.89% (95% CI: 40.89–62.9), with a level of heterogeneity (I^2^ = 99.55%, p < 0.001 (**Fig 2**).

### Publication bias and heterogeneity

**Fig 3:**
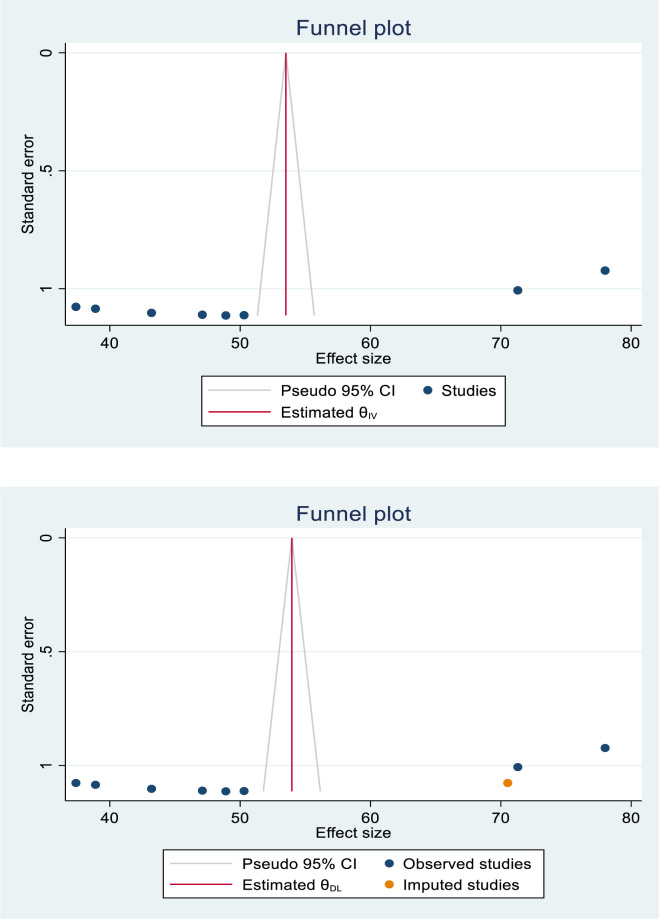
(A) Funnel plot to test publication bias of eight studies. (B) Result of trim and fill analysis for adjusting publication bias of the nine studies.

The funnel plot results were asymmetric, indicating the presence of publication bias among the studies included (**Fig 3A**). Egger’s regression test was done and revealed the presence of publication bias across studies (p-value = 0.0001). The Duval and Tweedie nonparametric trim and fill analysis was used to correct publication bias among the studies. As a result, publication bias was corrected when one missing study was filled in the funnel plot using trim and fill analysis. After the filling of one study, nine studies were included, and the trim and fill analysis gave the pooled modern contraceptive utilization of 53.96 (43.57–64.35) (**Fig 3B**).

### Sensitivity analysis

**Fig 4:**
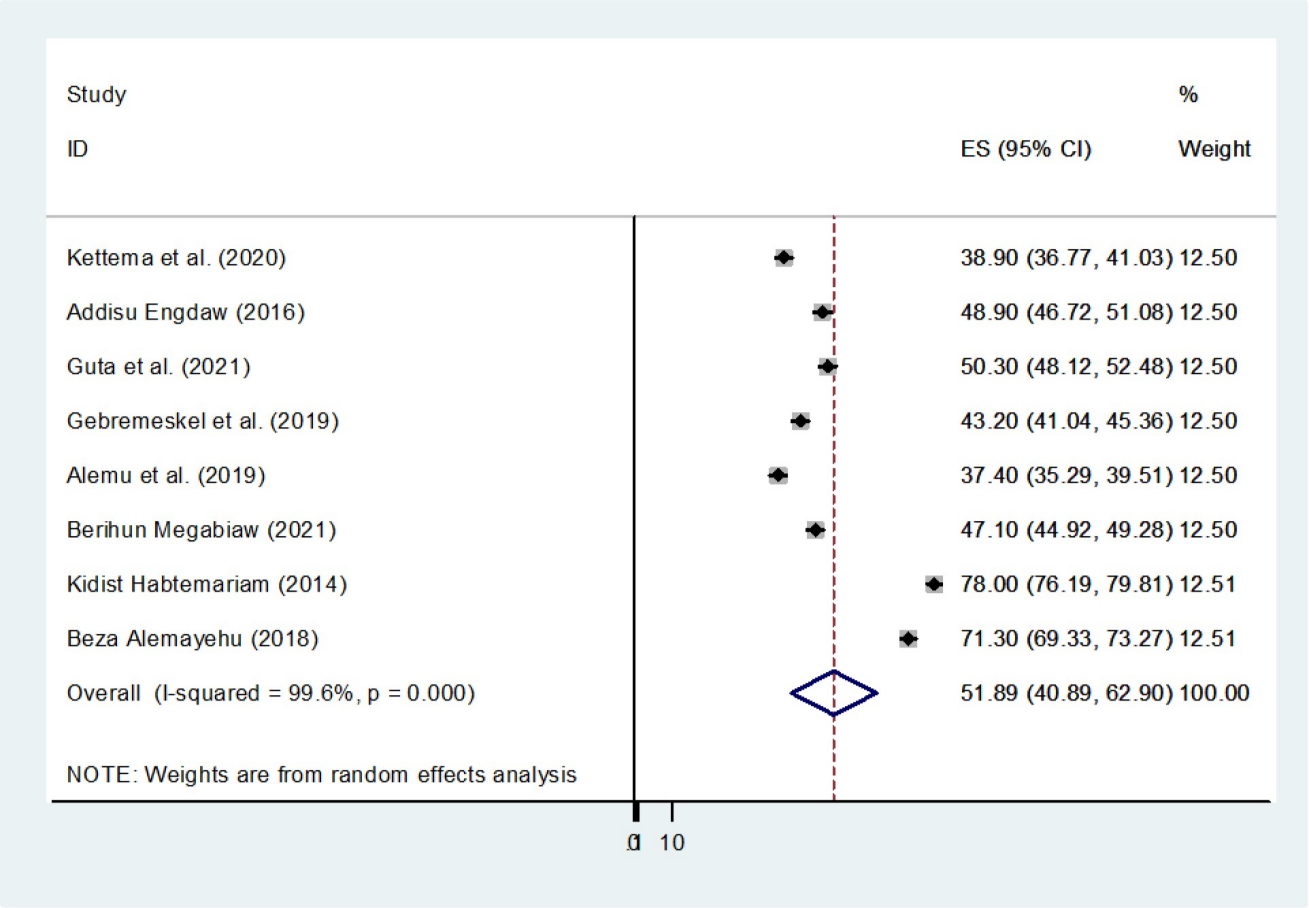
Sensitivity analysis of pooled modern contraceptive utilization in Ethiopia, 2023.

To identify the potential source of heterogeneity observed in the pooled modern contraceptive utilization, the authors conducted a leave-one-out sensitivity analysis. The result of the sensitivity analysis found that the findings did not rely on a particular study. The pooled modern contraceptive utilization varied and ranged from 37.4% (35.28, 39.51) to 78% (76.19, 79.81) after adding one study (**Fig 4**).

### Subgroup analysis

**Fig 5:**
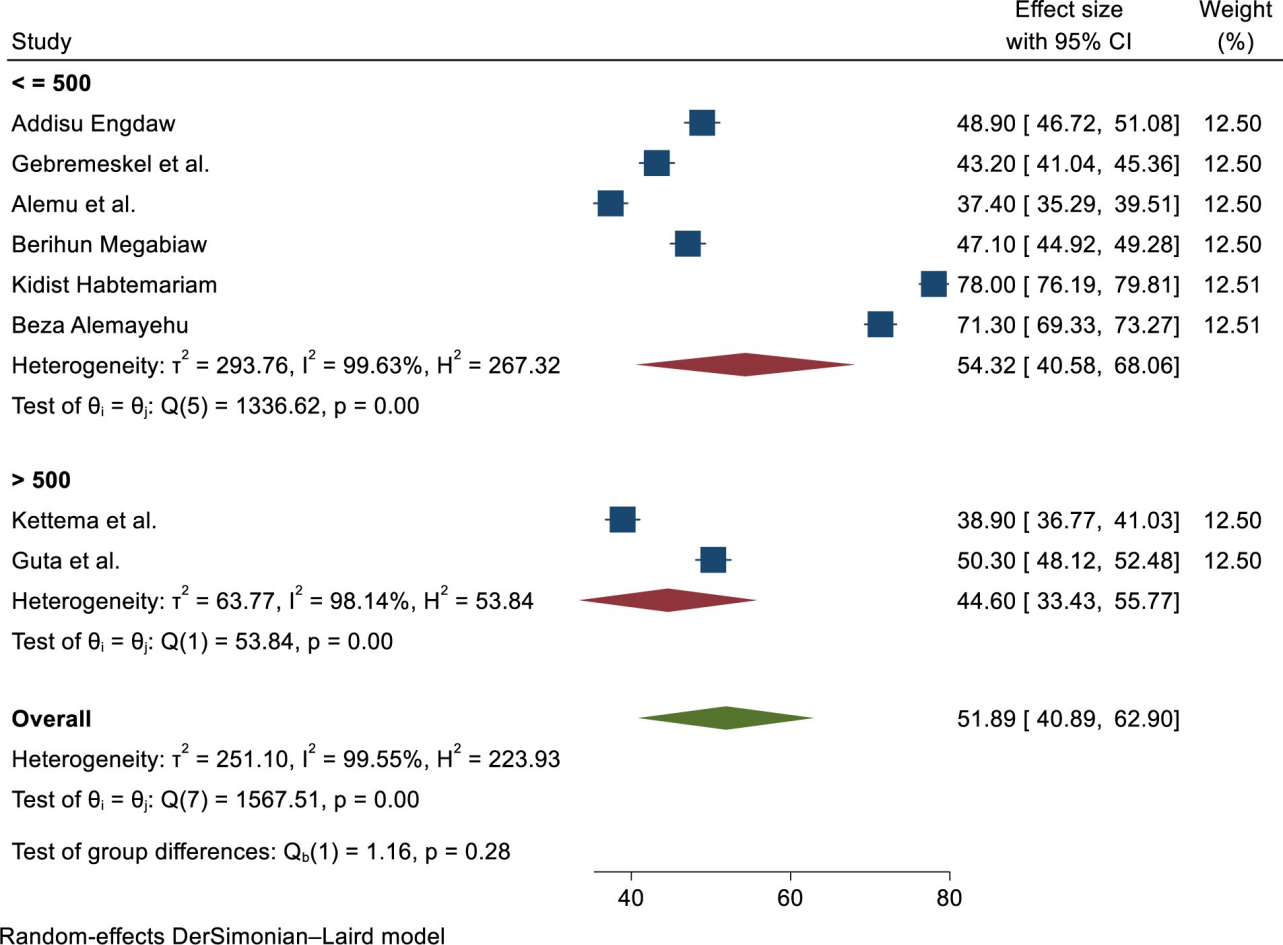
Sub-group analysis of modern contraceptive utilization in Ethiopia by sample size, 2023.

**Fig 6:**
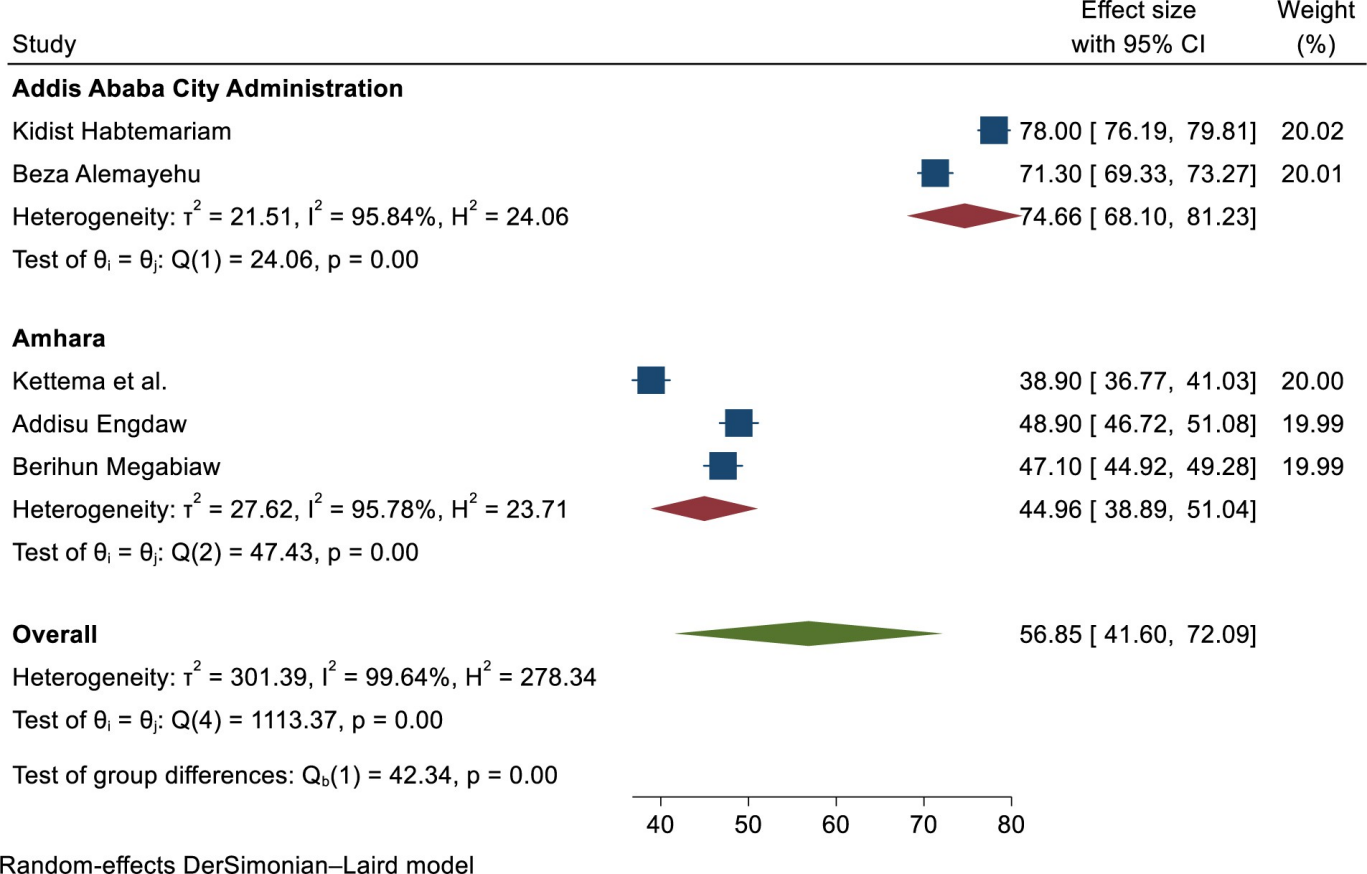
Sub-group analysis of modern contraceptive utilization in Ethiopia by region, 2023.

Subgroup analysis was conducted by region, and sample size and revealed substantial heterogeneity across the studies in terms of modern contraceptive utilization (I^2^ > 98.14%, P ≤ 0.001). Modern contraceptive utilization by region was found to be highest in the Addis Ababa city administration at 74.66% (68.1–81.23). In addition; the result of subgroup analysis was conducted by study sample size the highest utilization showed 54.32% (40.58–68.06) among sample sizes less or equal to five thundered respectively (**Figs 5 and 6**).

### Factors affecting modern contraceptive utilization

In this systematic review and meta-analysis; being married, facing a history of sexual assault/rape in the street life, aged between 25–35 years, getting advice from health professionals, having a history of pregnancy in street life, and having no more child wanted were significantly associated with modern contraceptive utilization associated significantly with significantly associated with modern contraceptive utilization.

**Fig 7:**
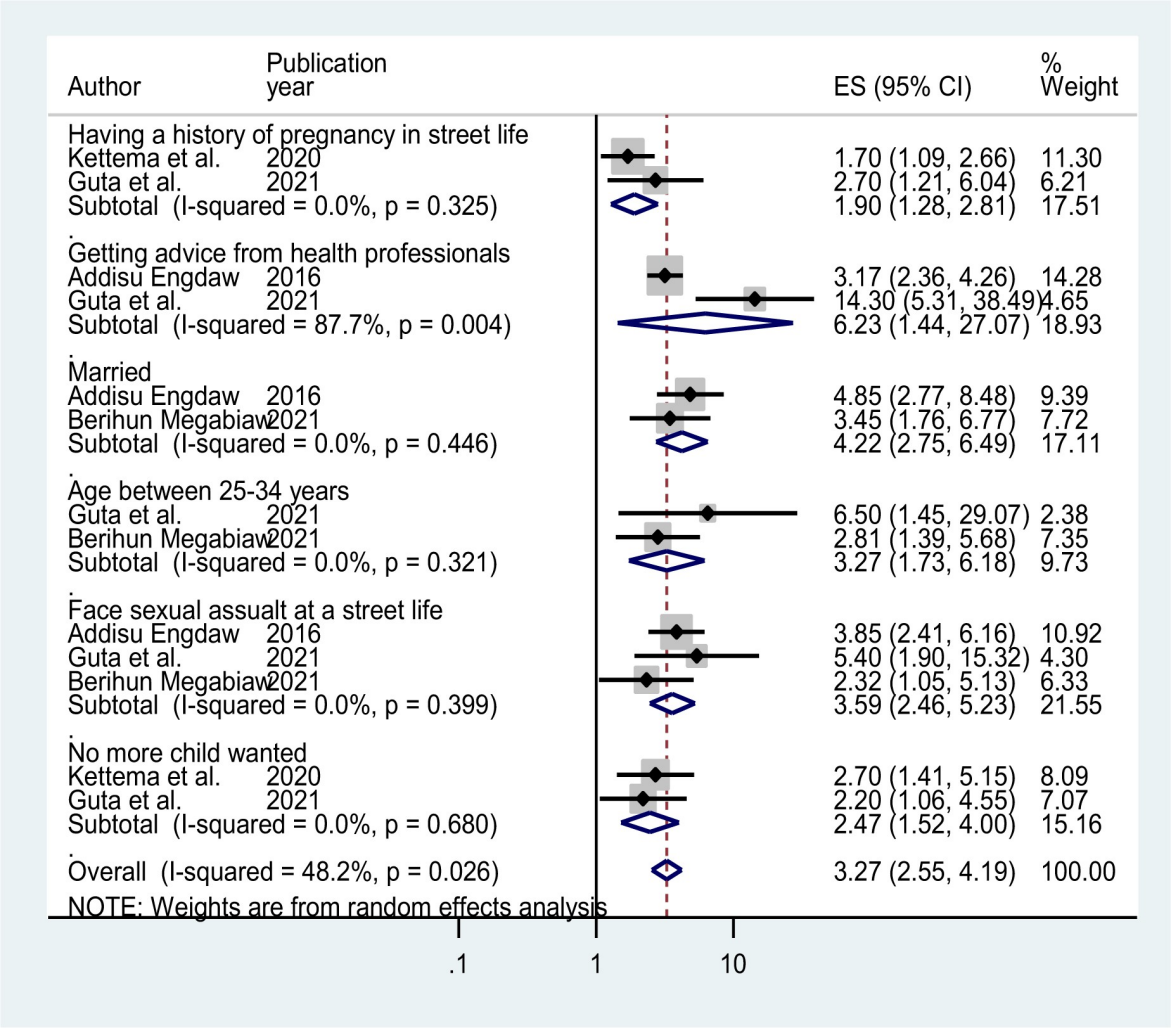
Factors associated with modern contraceptive utilization in Ethiopia, 2023.

In this review, two primary articles [22, 23] found that street women being married were more likely to utilize modern contraceptive methods (OR = 4.22, 95%CI, 2.75–6.49) than those counterparts. The pooled odds ratio was estimated using a random-effect model and no heterogeneity was observed between the studies, evidenced by (I^2^  =  0.00%, p  =  0.446). The pooled effect of three studies revealed that women who faced sexual assault/rape in the street were more likely to utilize modern contraceptive methods (OR = 3.59, 95%CI, 2.46–5.23) compared with their counterparts [4, 9, 25] (**Fig 7**).

Two studies found that ages between 25–35 years were more likely to utilize modern contraceptive methods (OR = 3.27, 95%CI, 1.73–6.18) than their counterparts. Getting advice from health professionals was strongly associated with the utilization of modern contraceptive methods (OR = 6.23, 95%CI, 1.44–27.07) as compared to not being advised by health professionals (**Fig 7**).

Women who had a history of pregnancy in street life increased almost two times the utilization of modern contraceptive methods (OR = 1.90, 95%CI, 1.28–2.81). Random-effect model analysis was conducted to estimate the pooled odds ratio, and no heterogeneity effect was observed across the studies (I^2^ = 0.00%, p = 0.325). The pooled effect of two studies revealed that no more children wanted increased two-folds of modern contraceptive utilization (OR = 2.47, 95%CI, 1.52–5.00). 7). Street-working women who did not want to have children in the future were two times more likely to use modern contraceptives as compared to those who wanted children within two years (**Fig 7**).

## Discussion

The purpose of this systematic review and meta-analysis was to identify the pooled modern contraceptive utilization and associated factors among street reproductive-age women in Ethiopia. In this study, the pooled prevalence of modern contraceptive utilization was 51.89%, with a 95% confidence interval of 40.89% - 62.9%. More than half of street reproductive-age women utilize modern contraceptive methods, but still not enough among such risk groups for unwanted pregnancy. This low utilization of modern contraceptives among street reproductive-age women might be due to several reasons lack of knowledge, sex education, and access to services; risk misperceptions; and ignorance might be a contributor [26].

Factors such as married street women, women who faced sexual assault or rape, ages between 25–35 years, getting advice from health professionals, having a history of pregnancy in street life, and having no more child wanted were associated with modern contraceptive utilization.

This could be because education is one of the most important factors in women’s empowerment and decision to use family planning services when compared to uneducated women. In addition, women who have been educated are more likely to use modern contraceptives, visit a health facility, and receive counseling or other family planning services. Furthermore, this may be because education for women aids in their understanding of their rights and responsibilities regarding sexual and reproductive health issues.

The likelihood of modern contraceptive use was higher among married street reproductive-age women compared to those who are unmarried category. This could be due to a desire to avoid additional suffering caused by unintended pregnancy and its consequences. Further, the possible explanation is that modern contraception is a safe and cost-effective method of preventing unintended pregnancy, spacing children, and limiting family size [27–29].

Street women who faced sexual assault or rape on a street life were more likely to use modern contraceptive methods when compared to those women who did not face such types of problems. This is similar to the study conducted in Colombia [29]. This is because unprotected sexual intercourse leads to unplanned or unwanted pregnancy, and the women are ready to use modern contraceptive methods. Also, women with a history of sexual assault or rape may be at risk, and they seek a solution such as emergency contraceptive pills to avoid unintended pregnancy and other problems.

This study also showed that women who have children after joining street life are two times more likely to utilize modern contraceptive methods. This might be street women who have a history of pregnancy after joining into street life and have faced the burden of unintended pregnancy and have become conscious and decided to utilize modern contraceptive methods. In addition, street who experience such problems leads to taking a lesson from previous sufferings of labor and childcare including feeding during difficult street life might be the possible reasons to utilize modern contraceptive methods [19].

The limitation of this study was a lack of articles to compare utilization among street reproductive-age women; the number of articles was small, making it difficult to generalize the findings; and the studies included in this review were limited to certain areas, suggesting that other regions may be underrepresented.

## Conclusions

Half of street-working reproductive-age women use modern contraceptive methods. The meta-analysis indicates that modern contraceptive utilization is associated with being married, facing a history of sexual assault/rape in street life, aging between 25–35 years, getting advice from health professionals, having a history of pregnancy in street life, and having no more child wanted. Every concerned body or stakeholder should be involved in awareness creation and implement health education programs for this marginalized and risky group to increase utilization to overcome reproductive health problems.

## Supporting information

S1 Table PRISMA checklist for modern contraceptive utilization in Ethiopia, 2023. (DOCX)

S2 Table A Searching strategy for modern contraceptive utilization and associated factors among street women in Ethiopia, 2023. (DOCX)

S3 Table Newcastle-Ottawa quality assessment scale for included studies to assess modern contraceptive utilization and associated factors among street women in Ethiopia, 2023. (DOCX)

S1 File Reason for exclusion of articles for utilization of modern contraceptive among street women in Ethiopia, 2023. (DOCX)
