# Supplementary material for: Modern contraceptive utilization and associated factors among street working reproductive age women in Ethiopia: A systematic review and meta-analysis
Source: PLoS One. 2024 Dec 27;19(12):e0312569. doi: 10.1371/journal.pone.0312569 (PMC11676527; doi:10.1371/journal.pone.0312569)
Supplement: S2 Table — (DOCX) [file pone.0312569.s002.docx]

**S2 Table:** Searching strategy for modern contraceptive utilization and associated factors among street women in Ethiopia, 2023..

| Databases | Searching terms | Number of studies |
| --- | --- | --- |
| PubMed | (Modern[All Fields] AND ("contraceptive agents"[All Fields] OR "contraceptive devices"[MeSH Terms] OR ("contraceptive"[All Fields] AND "devices"[All Fields]) OR "contraceptive devices"[All Fields] OR "contraceptive"[All Fields] OR "contraceptive agents"[MeSH Terms] OR ("contraceptive"[All Fields] AND "agents"[All Fields]) OR "contraceptive agents"[All Fields]) AND ("statistics and numerical data"[Subheading] OR ("statistics"[All Fields] AND "numerical"[All Fields] AND "data"[All Fields]) OR "statistics and numerical data"[All Fields] OR "utilization"[All Fields]) AND associated[All Fields] AND factors[All Fields] AND street[All Fields] AND ("women"[MeSH Terms] OR "women"[All Fields]) AND ("Ethiopia"[MeSH Terms] OR "Ethiopia"[All Fields]) | 182 |
| Google scholar | (Modern[All Fields] AND ("contraceptive agents"[All Fields] OR "contraceptive devices"[MeSH Terms] OR ("contraceptive"[All Fields] AND "devices"[All Fields]) OR "contraceptive devices"[All Fields] OR "contraceptive"[All Fields] OR "contraceptive agents"[MeSH Terms] OR ("contraceptive"[All Fields] AND "agents"[All Fields]) OR "contraceptive agents"[All Fields]) AND ("statistics and numerical data"[Subheading] OR ("statistics"[All Fields] AND "numerical"[All Fields] AND "data"[All Fields]) OR "statistics and numerical data"[All Fields] OR "utilization"[All Fields]) AND associated[All Fields] AND factors[All Fields] AND street[All Fields] AND ("women"[MeSH Terms] OR "women"[All Fields]) AND ("Ethiopia"[MeSH Terms] OR "Ethiopia"[All Fields]) | 26 |
| HINARI | Modern contraceptive utilization and associated factors among street women in Ethiopia | 96 |
| Others databases |  | 8 |
| Total retrieved |  | 312 |
| Included |  | 8 |
