# Supplementary material for: Modern contraceptive utilization and associated factors among street working reproductive age women in Ethiopia: A systematic review and meta-analysis
Source: PLoS One. 2024 Dec 27;19(12):e0312569. doi: 10.1371/journal.pone.0312569 (PMC11676527; doi:10.1371/journal.pone.0312569)
Supplement: S3 Table — (DOCX) [file pone.0312569.s003.docx]

**S3 Table:** Newcastle-Ottawa Quality Assessment Scale for observational studies to assess modern contraceptive utilization among street women in Ethiopia, 2023.

| Authors | Representatives | Sample size | Non -responders | Ascertainment | comparability | outcome | Quality score |
| --- | --- | --- | --- | --- | --- | --- | --- |
| Kettema et al.(2020) | 2 | 2 | 1 | 2 | 1 | 1 | 9 |
| Addisu Engdaw (2016) | 1 | 2 | 1 | 1 | 1 | 1 | 7 |
| Guta et al.(2021) | 2 | 2 | 1 | 2 | 1 | 1 | 9 |
| Gebremeskel et al.(2019) | 1 | 1 | 1 | 2 | 1 | 2 | 8 |
| Alemu et al.(2019) | 1 | 2 | 1 | 2 | 1 | 1 | 8 |
| Berihun Megabiaw (2021) | 2 | 2 | 1 | 1 | 1 | 2 | 9 |
| Kidist Habtemariam(2014) | 2 | 1 | 1 | 1 | 1 | 1 | 7 |
| Beza Alemayehu(2018) | 2 | 1 | 1 | 1 | 2 | 1 | 8 |

**Interpretation of the score**: Very Good Studies: 9-10 points, Good Studies: 7-8 points, Satisfactory Studies: 5-6 points, and Unsatisfactory Studies: 0 to 4 points
