# Supplementary material for: Modern contraceptive utilization and associated factors among street working reproductive age women in Ethiopia: A systematic review and meta-analysis
Source: PLoS One. 2024 Dec 27;19(12):e0312569. doi: 10.1371/journal.pone.0312569 (PMC11676527; doi:10.1371/journal.pone.0312569)
Supplement: S1 File — (DOCX) [file pone.0312569.s004.docx]

Reason for exclusion of articles for utilization of modern contraceptive among street women in Ethiopia, 2023.

| S.N | Author | Title | Reasons for exclusion |
| --- | --- | --- | --- |
| 1 | Comfort AB et al., 2021 | The association between men's family planning networks and contraceptive use among their female partners: an egocentric network study in Madagascar. | Wrong setting and population |
| 2 | Crawford EE et al., 2021 | Modern contraceptive use among unmarried girls aged 15-19 years in Southwestern Nigeria: results from a cross-sectional baseline survey for the Adolescent 360 (A360) impact evaluation. | Wrong setting and population |
| 3 | Tegegne TK et al., 2020 | Spatial variations and associated factors of modern contraceptive use in Ethiopia. | Wrong outcomes |
| 4 | Comfort AB et al., 2021 | Social and provider networks and women's contraceptive use: Evidence from Madagascar. | Wrong setting and population |
| 5 | Ajayi AI et al., 2018 | Maternal health care visits as predictors of contraceptive use among childbearing women in a medically underserved state in Nigeria. | Wrong setting |
| 6 | Edietah EE et al., 2018 | Contraceptive use and determinants of unmet need for family planning; a cross-sectional survey in the Northwest Region, Cameroon. | Wrong setting |
| 7 | Chebet JJ et al., 2015 | "Every method seems to have its problems"- Perspectives on side effects of hormonal contraceptives in Morogoro Region, Tanzania. | Wrong setting |
| 8 | Ajong AB et al., 2018 | Contraceptive method mix and preference: A focus on long-acting reversible contraception in Urban Cameroon. | Wrong setting |
| 9 | Atchison CJ et al., 2019 | Sexuality, fertility and family planning characteristics of married women aged 15 to 19 years in Ethiopia, Nigeria and Tanzania | Wrong setting and population |
| 10 | Debebe S et al., 2017 | Modern contraceptive methods utilization and associated factors among reproductive aged women in rural Dembia District, northwest Ethiopia: Community based cross-sectional study. | Wrong population |
| 11 | Nsanya MK et al., 2019 | Modern contraceptive use among sexually active women aged 15-19 years in North-Western Tanzania: results from the Adolescent 360 (A360) baseline survey. | Wrong setting |
| 12 | Degefa Hidru H et al., 2020 | Modern Contraceptive Utilization and Its Associated Factors among Indigenous and Nonindigenous Married Women of Reproductive Age Group in Jigjiga Town, Eastern Ethiopia, 2018. | Wrong population |
| 13 | Debelew GT, and Habte MB, 2021 | Contraceptive Method Utilization and Determinant Factors among Young Women (15-24) in Ethiopia: A Mixed-Effects Multilevel Logistic Regression Analysis of the Performance Monitoring for Action 2018 Household Survey. | Wrong population |
| 14 | Solomon S et al., 2022 | Contraceptive Utilization and Unmet Need for Contraception Among Women Undergoing Treatment for Tuberculosis in Addis Ababa, Ethiopia: a Cross-Sectional Study. | Wrong population |
| 15 | Long JE et al., 2019 | Prevalence and predictors of unmet contraceptive need in HIV-positive female sex workers in Mombasa, Kenya. | Wrong setting |
| 16 | Yimer AS, and Modiba LM, 2019 | Modern contraceptive methods knowledge and practice among blind and deaf women in Ethiopia. A cross-sectional survey. | Wrong population |
| 17 | Mare KU et al., 2022 | Individual and community-level determinants of non-use of contraceptive among women with no fertility desire in Ethiopia: a multilevel mixed-effect analysis. | Wrong population |
| 18 | Fruhauf T et al., 2018 | Measuring family planning quality and its link with contraceptive use in public facilities in Burkina Faso, Ethiopia, Kenya and Uganda. | Wrong outcomes |
| 19 | Zerihun T et al, 2021 | Family planning for women with severe mental illness in rural Ethiopia: a qualitative study. | Wrong study design |
| 20 | Terefe G et al., 2022 | Unmet Need for Family Planning Service and Associated Factors Among Homeless Women of Reproductive Age Group in Jimma Zone Administrative Towns, Ethiopia. | Wrong outcomes |
| 21 | Mekonnen AG et al., 2022 | Determinants of adolescents' contraceptive uptake in Ethiopia: a systematic review of literature. | Wrong study design |
| 22 | Liu Z et al., 2021 | Identify Key Determinants of Contraceptive Use for Sexually Active Young People: A Hybrid Ensemble of Machine Learning Methods. | Wrong setting |
| 23 | Mardi A et al., 2018 | Factors influencing the use of contraceptives through the lens of teenage women: a qualitative study in Iran. | Wrong setting |
| 24 | Liu J et al., 2018 | Introducing the subcutaneous depot medroxyprogesterone acetate injectable contraceptive via social marketing: lessons learned from Nigeria's private sector. | Wrong setting |
| 25 | Mersha AG et al., 2019 | Contraceptive use among HIV-positive and negative women: implication to end unintended pregnancy. | Wrong population |
| 26 | Slaymaker E et al., 2020 | Trends in sexual activity and demand for and use of modern contraceptive methods in 74 countries: a retrospective analysis of nationally representative surveys. | Wrong population and setting |
| 27 | Le Voir R, 2022 | Measuring contraceptive use in a displacement-affected population using the Multiple Indicator Cluster Survey: The case of Iraq. | Wrong setting |
| 28 | Capurchande R et al., 2016 | "It is challenging… oh, nobody likes it!": a qualitative study exploring Mozambican adolescents and young adults' experiences with contraception. | Wrong setting |
| 29 | Mesfin Yesgat Y et al., 2020 | Utilization of Family Planning Methods and Associated Factors Among Reproductive-Age Women with Disability in Arba Minch Town, Southern Ethiopia. | Wrong population |
| 30 | Stifani BM et al., 2018 | From Pill to Condom, or Nothing at all: HIV Diagnosis and Discontinuation of Highly Effective Contraceptives Among Women in Northeast Brazil. | Wrong setting |
| 31 | Ajayi AI et al., 2018 | Use of traditional and modern contraceptives among childbearing women: findings from a mixed methods study in two southwestern Nigerian states. | Wrong setting |
| 32 | Bwambale MF et al., 2022 | Rural-Urban Migration, Childbearing Decision-Making, Fertility and Contraceptive Perspectives of Street Adolescents and Youth in Kampala, Uganda. | Wrong setting |
| 33 | Mukamuyango J et al., 2020 | Uptake of long-acting reversible contraception following integrated couples HIV and fertility goal-based family planning counselling in Catholic and non-Catholic, urban and rural government health centers in Kigali, Rwanda. | Wrong setting |
| 34 | Singh P et al., 2020 | The levels and trends of contraceptive use before first birth in India (2015-16): a cross-sectional analysis. | Wrong setting |
| 35 | Idowu A et al., 2020 | Modern Contraception: Uptake and Correlates among Women of Reproductive Age-Group in a Rural Community of Osun State, Nigeria. | Wrong setting |
| 36 | Bukenya JN et al., 2019 | Contraceptive use, prevalence and predictors of pregnancy planning among female sex workers in Uganda. | Wrong setting |
| 37 | Nkoka O et al., 2020 | Multilevel analysis of factors associated with unmet need for family planning among Malawian women. | Wrong setting |
| 38 | Nuwasiima A et al., 2019 | Acceptability and utilization of family planning benefits cards by youth in slums in Kampala, Uganda. | Wrong setting |
| 39 | Tang JH et al., 2016 | Association between contraceptive implant knowledge and intent with implant uptake among postpartum Malawian women. | Wrong setting |
| 40 | Smith C et al., 2019 | Characteristics and contraceptive outcomes of women seeking medical or surgical abortion in reproductive health clinics in Cambodia. | Wrong setting |
| 41 | Ajayi AI et al., 2017 | Unplanned pregnancy-risks and use of emergency contraception: a survey of two Nigerian Universities. | Wrong setting |
| 42 | Ehiaghe AD, and Barrow A., 2022 | Parental Knowledge, Willingness, and Attitude towards Contraceptive Usage among Their Unmarried Adolescents in Ekpoma, Edo State, Nigeria. | Wrong setting |
| 43 | Ajong AB et al., 2016 | Determinants of unmet need for family planning among women in Urban Cameroon: a cross-sectional survey in the Biyem-Assi Health District, Yaoundé. | Wrong setting |
| 44 | Adeyemi AS et al., 2016 | Contraceptive prevalence and determinants among women of reproductive age group in Ogbomoso, Oyo State, Nigeria. | Wrong setting |
| 45 | Orach CG et al., 2015 | Perceptions, attitude and use of family planning services in post conflict Gulu district, northern Uganda. | Wrong setting |
| 46 | Salifu MG, and Mohammed K, 2020 | Prevalence and Predictors of Contraceptives Use among Women Aged (15-49 years) with Induced Abortion History in Ghana. | Wrong setting |
| 47 | Castro et al., 2021 | Is the use of contraceptives associated with periodontal diseases? | Wrong study design |
| 48 | Rouncivell, L., 2020 | Knowledge, attitudes and perceptions of long-acting reversible contraceptive (LARC) methods among healthcare workers in sub-Saharan Africa. | Wrong study design |
| 49 | Tang JH et al., 2012 | Hormonal and intrauterine methods for contraception for women aged 25 years and younger. | Wrong setting |
| 50 | Burke HM et al., 2022 | Reproductive empowerment and contraceptive self-care. | Wrong study design |
| 51 | Cherie N et al., 2022 | Post Abortion Contraceptive Use and Determinant Factors in Ethiopia. | Wrong study design |
| 51 | Fenta SM, and Gebremichael SG, 2021 | Predictors of modern contraceptive usage among sexually active rural women in Ethiopia. | Wrong Population |
| 52 | Gebremeskel F et al., 2017 | Prevalence of modern contraceptive utilization and associated factors among women of reproductive age group at Boditi Town, Wolayita Zone, SNNPR, Ethiopia. | Wrong outcomes |
| 53 | Meselu W et al., 2022 | Trends and predictors of modern contraceptive use among married women: Analysis of 2000–2016 Ethiopian Demographic and Health Surveys. | Wrong population |
| 54 | Hailegebreal S et al., 2021 | Individual and community-level factors associated with modern contraceptive use among adolescent girls and young women in Ethiopia: a multilevel analysis of 2016 Ethiopia demographic and health survey. | Wrong outcomes |
| 55 | Belete N et al., 2018 | Prevalence and factors associated with modern contraceptive discontinuation among reproductive age group women, a community based cross-sectional study in Humera town, northern Ethiopia. | Wrong population |
| 56 | Tsegay K et al., 2017 | Modern contraceptive methods utilization and associated factors among women with disabilities in Mekelle Town, Tigray, Ethiopia. | Wrong population |
| 57 | Lakew Y et al., 2013 | Geographical variation and factors influencing modern contraceptive use among married women in Ethiopia. | Wrong population and outcome |
| 58 | Tessema ZT et al., 2021 | Pooled prevalence and determinants of modern contraceptive utilization in East Africa. | Wrong setting |
| 59 | Zegeye B et al., 2021 | Modern contraceptive utilization and its associated factors among married women in Senegal. | Wrong setting |
| 60 | Alemu L et al., 2020 | Contraceptive use and associated factors among women seeking induced abortion in Debre Marko’s town, Northwest Ethiopia. | Wrong population |
| 61 | Seyife A et al., 2019 | Utilization of modern contraceptives and predictors among women in Shimelba refugee camp, Northern Ethiopia. | Wrong population |
| 62 | Gejo NG et al., 2019 | Postpartum modern contraceptive use and associated factors in Hossana town. | Wrong population |
| 63 | Tesema ZT et al., 2022 | Determinants of modern contraceptive utilization among married women in sub-Saharan Africa. | Wrong setting |
| 64 | Shiferaw K, and Musa A, 2017 | Assessment of utilization of long-acting reversible contraceptive and associated factors among women of reproductive age in Harar City, Ethiopia. | Wrong population |
| 65 | Melaku YA, and Zeleke EG, 2014 | Contraceptive utilization and associated factors among HIV positive women on chronic follow up care in Tigray Region, Northern Ethiopia. | Wrong population |
| 66 | Tsehaye WT et al., 2011 | Assessment of modern contraceptive methods utilization and its determinant factors among women of reproductive age groups at Shire Endaslasie town, Tigray, northern Ethiopia. | Wrong population |
| 67 | Akoth C et al., 2021 | Factors associated with the utilisation and unmet need for modern contraceptives among urban women in Kenya. | Wrong setting |
| 68 | Girma T et al., 2016 | Prevalence and factors influences utilization of modern contraceptive methods among married women of reproductive age group (15-49 Years) in Holeta town, Oromia, Ethiopia. | Wrong population |
| 69 | Seidu AA et al., 2020 | Modern contraceptive utilization and associated factors among married and cohabiting women in Papua New Guinea. | Wrong setting |
| 70 | Shaweno T, and Kura Z, 2020 | Determinants of modern contraceptive use among sexually active men in Ethiopia; using EDHS 2016 national survey. | Wrong population |
| 71 | Ahinkorah BO et al., 2021 | Factors associated with modern contraceptive use among women with no fertility intention in sub-Saharan Africa: evidence from cross-sectional surveys of 29 countries. | Wrong setting |
| 72 | Tewabe T et ai., 2020 | Contraceptive use and associated factors among sexually active reproductive age HIV positive women attending ART clinic at Felege Hiwot Referral Hospital, Northwest Ethiopia. | Wrong population |
| 73 | Gebremariam A, and Gebremariam H, 2017 | Contraceptive use among lactating women in Ganta-Afeshum District, Eastern Tigray, Northern Ethiopia, 2015. | Wrong population |
| 74 | Yesuf KA et al., 2020 | Geographical variation and factor associated with modern contraceptive utilization among Young married women aged between 15-24 years: Spatial and multilevel analysis of EDHS 2016. | Wrong population |
| 75 | Mekonnen Bd ET AL., 2021 | Knowledge and associated factors of postpartum contraceptive use among women in the extended postpartum period in Gondar city, Northwest Ethiopia. | Wrong outcomes |
| 76 | Demissie DB, and Dadi HW, 2021 | Immediate postpartum modern family planning utilization and associated factors among women gave birth public health facilities, Addis Ababa, Ethiopia. | Wrong population |
| 77 | Zerihun T et al., 2020 | Family planning awareness, utilization and associated factors among women of reproductive age attending psychiatric outpatient care, a cross-sectional study, Addis Ababa, Ethiopia. | Wrong population |
| 78 | Weldemariam KT et al., 2019 | Reasons and multilevel factors associated with unscheduled contraceptive use discontinuation in Ethiopia: evidence from Ethiopian demographic and health survey 2016. | Wrong outcomes |
| 79 | Tafa L, and Worku Y, 2021 | Family planning utilization and associated factors among postpartum women in Addis Ababa, Ethiopia, 2018. | Wrong population |
| 80 | Bitew F, and Nyarko SH, 2019 | Modern contraceptive use and intention to use; implication for under-five mortality in Ethiopia. | Wrong outcomes |
| 81 | Asresie MB et al., 2020 | Contraceptive use among women with no fertility intention in Ethiopia. | Wrong population |
| 82 | Wassihun B et al., 2021 | Prevalence of postpartum family planning utilization and associated factors among postpartum mothers in Arba Minch town, South Ethiopia. | Wrong population |
| 83 | Mahande MJ et al., 2020 | Factors associated with modern contraceptives use among postpartum women in Bukombe district, Geita region, Tanzania. | Wrong setting |
| 84 | Walelign D et al., 2014 | Modern contraceptive use among orthodox Christian and Muslim women of reproductive age group in Bahir Dar City, Northwest Ethiopia: comparative cross-sectional study. | Wrong outcomes |
| 85 | Bekere S et al., 2022 | Time to use modern contraceptives and associated factors among women of reproductive age in Ethiopia: survival analysis of the 2016 Ethiopian demographic and health survey data. | Wrong outcomes |
| 86 | Gebremichael H et al., 2014 | Acceptance of long-acting contraceptive methods and associated factors among women in Mekelle city, Northern Ethiopia. | Wrong outcomes |
| 87 | Aychew EW et al., 2022 | Utilization of long-acting contraceptive methods and associated factors among married women in Farta Woreda, Northwest Ethiopia: a community-based mixed method study. | Wrong population |
| 88 | Hidru HD et al., 2020 | Modern Contraceptive Utilization and Its Associated Factors among Indigenous and Nonindigenous Married Women of Reproductive Age Group in Jigjiga Town, Eastern Ethiopia, 2018. | Wrong population |
| 89 | Yalew M et al., 2020 | Individual and community-level factors associated with unmet need for contraception among reproductive-age women in Ethiopia; a multi-level analysis of 2016 Ethiopia Demographic and Health Survey. | Wrong population |
| 90 | Habtamu A et al., 2019 | Determinants of long-acting contraceptive utilization among married women of reproductive age in Aneded district, Ethiopia: a case-control study. | Wong population |
| 91 | Girma Garo M et al., 2021 | Unmet need for family planning and associated factors among currently married women of reproductive age in Bishoftu town, Eastern Ethiopia. | Wrong population |
| 92 | Aradom HS et al., 2020 | Factors associated with modern family planning use among women living with HIV who attended care and treatment clinics in Jigjiga town, Eastern Ethiopia. | Wrong population |
| 93 | Stonehill A et al., 2020 | Factors associated with long-acting and short-acting reversible contraceptive use in Ethiopia: an analysis of the 2016 Demographic and Health Survey. | Wrong outcomes |
| 94 | Ebrahim OA et al., 2021 | Geographic variation and associated factors of long-acting contraceptive use among reproductive-age women in Ethiopia. | Wrong outcomes |
| 95 | Haile D, and Lagebo B, 2022 | Magnitude of dual contraceptive method utilization and the associated factors among women on antiretroviral treatment in Wolaita zone, Southern Ethiopia. | Wrong population |
| 96 | Bekele D et al., 2021 | Contraceptive prevalence rate and associated factors among reproductive age women in four emerging regions of Ethiopia: a mixed method study. | Wrong population |
| 97 | Animen S et al., 2018 | Utilization of intra uterine contraceptive device and associated factors among reproductive age group of family planning users in Han Health Center, Bahir Dar, Northwest Amhara, Ethiopia, 2018. | Wrong outcomes |
| 98 | Terefe A, and Larson CP, 1993 | Modern contraception use in Ethiopia: does involving husbands make a difference? | Wrong publication date |
| 99 | Haile D, and Lagebo B, 2022 | Magnitude of dual contraceptive method utilization and the associated factors among women on antiretroviral treatment in Wolaita zone, Southern Ethiopia. Heliyon. 2022 Jun 1;8(6). | Wrong population |
| 100 | Gujo AB, and Kare AP, 2021 | Utilization of long-acting reversible contraceptives and associated factors among reproductive age women attending governmental health institutions for family planning services in Wondo Genet District, Sidama, National Regional State, Southern Ethiopia. | Wrong population |
| 101 | Abdalla KK, 2020 | Prevalence of and factors associated with modern contraceptive use among female sex workers in Dar Es Salaam, Tanzania. | Wrong setting |
| 102 | Kassie GM et al., 2014 | Assessment of pattern and determinants of contraceptive use among females of reproductive age in Kelala Town, Northern Ethiopia. | Wrong population |
| 103 | Demissie DB, and Gudisa T, 2019 | Dual contraceptive use and associated factors among women living with HIV attending art clinics in West Zone Health Facilities Oromia, Ethiopia. | Wrong population and outcomes |
| 104 | Tadele A, and Berhanu M, 2021 | Trends and factors influencing long-acting contraceptive utilisation among contraceptive users in Ethiopia. | Wrong population |
| 105 | Alemayehu SS et al., 2021 | Contraceptive Utilization and Unmet Need for Contraception Among Women Undergoing Treatment for Tuberculosis in Addis Ababa, Ethiopia; a Cross-sectional Study. | Wrong population |
| 106 | Zelalem D et al., 2021 | Association of Effective Spousal Family Planning Communication with couples’ modern contraceptive use in Harar, eastern Ethiopia. | Wrong outcomes |
| 107 | Tegegne BD et al., 2022 | Women’s intention to use long acting and permanent contraceptive methods and associated factors among family planning users in Addis Ababa, Ethiopia. | Wrong outcomes |
| 108 | Getahun DS et al., 2018 | Utilization and determinants of long term and permanent contraceptive methods among married reproductive age women at Janamora district, northwest Ethiopia. | Wrong population |
| 109 | Ntenda PA, 2016 | Factors associated with contraceptive use and intention to use contraceptives among married women in Ethiopia. | Wrong outcomes |
| 110 | Girma S, 2017 | Modern family planning Utilization among married rural Women in Ethiopia: the case of Sululta Woreda, Oromia special zone. | Wrong population |
| 111 | Enden MR et al., 2021 | Providing universal access to modern contraceptive methods: An extended cost-effectiveness analysis of meeting the demand for modern contraception in Ethiopia. | Wrong outcomes |
| 112 | Njotang PN et al., 2017 | Determinants of modern contraceptive practice in Yaoundé-Cameroon: a community based cross sectional study. | Wrong setting |
| 113 | Dake SK, and Abiso TL, 2020 | Reversible Long Term Contraceptives Utilization among Married Women of Reproductive Age Group in Areka Town, Southern Ethiopia. | Wrong population |
| 114 | Wulifan JK et al., 2017 | Factors associated with contraceptive use among women of reproductive age in rural districts of Burkina Faso. | Wrong setting |
| 115 | Rutaremwa G et al., 2015 | Predictors of modern contraceptive use during the postpartum period among women in Uganda: a population-based cross-sectional study. | Wrong population |
| 116 | Gonie A et al., 2018 | Determinants of family planning use among married women in bale eco-region, Southeast Ethiopia. | Wrong population |
| 117 | Tilahun D et al., 2010 | Predictors of emergency contraceptive use among regular female students at Adama University, Central Ethiopia. | Wrong population |
| 118 | Kebede F, 1989 | Characteristics influencing usage of modern contraception. | Wrong outcomes |
| 119 | Geda YF et al., 2021 | Immediate postpartum intrauterine contraceptive device utilization and influencing factors in Addis Ababa public hospitals: a cross-sectional study. | Wrong outcomes |
| 120 | Geleta D et al., 2021 | Prevalence and Predictors of Contraceptive Use Among Women of Premenopausal Period in Ethiopia. | Wrong population |
| 121 | Prata N et al., 2013 | Contraceptive use among women seeking repeat abortion in Addis Ababa, Ethiopia. | Wrong population |
| 122 | Olagunju OS et al., 2020 | Knowledge and factors associated with modern contraceptive use among young women in West African countries. | Wrong setting |
| 123 | Woldeyohannes D et al., 2022 | Reasons for low utilization of intrauterine device utilisation amongst short term contraceptive users in Hossana town, Southern Ethiopia: a qualitative study. | Wrong study Design |
| 124 | Lasong J et al., 2020 | Determinants of modern contraceptive use among married women of reproductive age: a cross-sectional study in rural Zambia. | Wrong setting |
| 125 | Bereku T et al., 2022 | Magnitude and factors for method discontinuation and switching among long-acting reversible contraceptive users in health facilities of Southern Ethiopia. | Wrong population |
| 126 | Kapadia-Kundu N et al., 2022 | Applying a gender lens to social norms, couple communication and decision making to increase modern contraceptive use in Ethiopia, a mixed methods study. | Wrong outcomes |
| 127 | Kalayu H, 2017 | Dual Contraceptive Use and Associated Factors among HIV Positive Women on Art Follow up In Mekelle Town Tigray, Ethiopia. | Wrong population |
| 128 | Emiru AA et al., 2020 | The role of maternal health care services as predictors of time to modern contraceptive use after childbirth in Northwest Ethiopia: Application of the shared frailty survival analysis. | Wrong outcomes |
| 129 | Tadesse D et al., 2022 | Pregnancy and sexual related problems among women living on the street at Dire Dawa City, Ethiopia 2021. | Wrong outcomes |
| 130 | Gurmu E, and Mturi AJ, 2013 | Trend and correlates of contraceptive use in rural and urban Ethiopia; is there a link to the health extension programme. | Wrong outcomes |
| 131 | Maleche DA, 2020 | Determinants of unmet need for modern contraception among women of reproductive age living in Eldoret town, kenya | Wrong setting |
| 132 | Ajayi AI et al., 2018 | Use of traditional and modern contraceptives among childbearing women: findings from a mixed methods study in two southwestern Nigerian states. | Wrong setting |
| 133 | Mersha AG et al., 2019 | Contraceptive use among HIV-positive and negative women: implication to end unintended pregnancy. | Wrong population |
| 135 | Nega G et al., 2021 | Discontinuation rate and associated factors among contraceptive implant users in Kersa district, southwestern Ethiopia. | Wrong outcomes |
| 136 | Sufa A et al., 2013 | Utilization of family planning methods and associated factors among women living with HIV attending ART clinics in Nekemte Public Health Facilities, East Wollega Zone, Ethiopia. | Wrong population |
| 137 | Ayele AD et al., 2021 | Dual contraceptive utilization and determinant factors among HIV positive women in Ethiopia. | Wrong population |
| 138 | Mihretie GS et al., 2022 | Factors associated with discontinuation among long-acting reversible contraceptive users: a multisite prospective cohort study in urban public health facilities in Ethiopia. | Wrong population |
| 139 | Syum H et al., 2019 | Intention to use long-acting and permanent contraceptive methods and associated factors in health institutions of Aksum Town, North Ethiopia. | Wrong outcomes |
| 140 | Tareke AA et al., 2022 | Trends and predictors of change of unmet need for family planning among reproductive age women in Ethiopia. | Wrong outcomes |
| 141 | Osinowo K et al., 2020 | Patterns of triggers, ideation and motivational factors of contraceptive utilization among women and gate-keepers in Nigeria. | Wrong setting and study design |
| 142 | Ahinkorah BO, 2020 | Predictors of modern contraceptive use among adolescent girls and young women in sub-Saharan Africa. | Wrong setting |
| 143 | Tafa M, and Haidar J, 2014 | Effect of modern family planning use on nutritional status of women of reproductive age group at Tena District, Arsi Zone, Oromiya Region, Ethiopia. | Wrong outcomes |
| 144 | Wahed T et al., 2017 | Knowledge, use and associated factors relating to modern contraceptive methods among female sex workers in Dhaka, Bangladesh. | Wrong setting |
| 145 | Sedlander E et al., 2018 | Understanding modern contraception uptake in one Ethiopian community: a case study. | Wrong outcomes |
| 146 | Anik AI et al., 2022 | Association between socioeconomic factors and unmet need for modern contraception among the young married women. | Wrong setting |
| 147 | Matungulu MC et al., 2017 | Factors associated with the use of modern contraceptive methods by women in Marital union in the city of Lubumbashi, Democratic Republic of Congo. | Wrong setting |
| 148 | Ewerling F et al., 2021 | Modern contraceptive use among women in need of family planning in India. | Wrong setting |
| 149 | Some SY et al., 2021 | Empowerment and use of modern contraceptive methods among married women in Burkina Faso. | Wrong setting |
| 150 | Osaro BO et al., 2017 | Knowledge of modern contraceptives and their use among rural women of childbearing age in Rivers State Nigeria. | Wrong setting |
| 151 | Wasswa R et al., 2021 | Multilevel mixed effects analysis of individual and community level factors associated with modern contraceptive use among married women in Uganda. | Wrong setting |
| 152 | Kiondo KS et al., 2020 | Prevalence and factors associated with postpartum use of long-acting reversible contraception in Bukombe District, Geita Region, Tanzania. | Wrong setting |
| 153 | Tesfay F et al., 2018 | Resumption of Postpartum Sexual Intercourse and Use of Modern Contraceptive among In-Union Women in Addis Ababa. | Wrong outcomes |
| 154 | Shitu K et al., 2022 | Individual and community-level determinants of intention to use contraceptive among married women in Ethiopia. | Wrong population |
| 155 | Chantal UM et al., 2020 | Prevalence and factors associated with modern contraceptive methods dropout among women of reproductive age in Gatsibo district in Rwanda. | Wrong outcomes |
| 156 | Martin V et al., 2019 | Prevalence and determinants of modern contraceptive methods use among women of reproductive age (15-49 years) in rural setting: a case of Kishapu District, Shinyanga Region. | Wrong setting |
| 157 | Hailu S et al., 2022 | Unmet need for contraception among married adolescent girls and young women in Haramaya health and demographic surveillance system, Eastern Ethiopia. | Wrong outcomes |
| 158 | Forty J et al., 2021 | Patterns and determinants of modern contraceptive use and intention to use contraceptives among Malawian women of reproductive ages (15–49 years). | Wrong setting |
| 159 | Edietah EE et al., 2018 | Contraceptive use and determinants of unmet need for family planning; a cross-sectional survey in the Northwest Region, Cameroon. | Wrong setting |
| 160 | Yabesera M, 2020 | Proportion and Associated Factors of Delayed Pregnancy Return Following Discontinuation of Reversible Modern Contraceptives among Pregnant Women Attending Antenatal Care at Public Health Facilities in Bahir Dar, Amhara Region, Ethiopia. | Wrong outcomes |
| 161 | Temesgen K et al., 2017 | Assessment of knowledge, attitude and practice towards emergency contraceptives and associated factors among Wollo University (Dessie Campus) undergraduate female students in Dessie, Ethiopia. | Wrong population |
| 162 | Kamal SM, 2015 | Socioeconomic factors associated with contraceptive use and method choice in urban slums of Bangladesh. | Wrong setting |
| 163 | Tadele A et al., 2019 | Predictors of unmet need for family planning among all women of reproductive age in Ethiopia. | Wrong outcomes |
| 164 | Alawode OA et al., 2022 | Prevalence and determinants of intention to use modern contraceptives among grand-multiparous women in sub-Saharan Africa. | Wrong setting |
| 165 | Weldegebreal R et al., 2015 | Unintended pregnancy among female sex workers in Mekelle city, northern Ethiopia: a cross-sectional study. | Wrong population |
| 166 | Chaudhari A et al., 2022 | Factors Associated with Utilization of Modern Family Planning Methods among Married Women of Tharu Community of Madhuwan Municipality in Bardiya District of Nepal. | Wrong setting |
| 167 | Ebeny FT, 2017 | Assessment of Factors Affecting Modern Contraceptive Use Among Female Traders. | Wrong setting |
| 168 | John NA et al., 2020 | Quality of contraceptive use and women’s paid work and earnings in Peri-Urban Ethiopia. | Wrong outcomes |
| 169 | Decker M, and Constantine NA, 2011 | Factors associated with contraceptive use in Angola. | Wrong setting |
| 170 | Rwabilimbo MM et al., 2021 | Initiation of postpartum modern contraceptive methods: evidence from Tanzania demographic and health survey. | Wrong setting |
| 171 | Gebre MN, and Edossa ZK, 2020 | Modern contraceptive utilization and associated factors among reproductive-age women in Ethiopia. | Wrong population |
| 172 | Debebe S et al., 2017 | Modern contraceptive methods utilization and associated factors among reproductive aged women in rural Dembia District, northwest Ethiopia. | Wrong outcomes |
| 173 |  |  |  |
| 174 | Worku AG et al., 2015 | Trends of modern contraceptive use among young married women based on the 2000, 2005, and 2011 Ethiopian demographic and health surveys. | Wrong outcomes |
| 175 | Belda SS et al., 2017 | Modern contraceptive utilization and associated factors among married pastoralist women in Bale eco-region, Bale Zone, Southeast Ethiopia. | Wrong population |
| 176 | Nibret Mihretie G et al., 2020 | Postpartum modern contraceptive utilization and associated factors among women who gave birth in the last 12 months in Addis Zemen, South Gondar, Ethiopia. | Wrong population |
| 177 | Teshome L et al., 2021 | Modern contraceptives use and associated factors among adolescents and youth in Ethiopia. | Wrong population |
| 178 | Wandera SO et al., 2018 | Intimate partner violence and current modern contraceptive use among married women in Uganda. | Wrong setting |
| 179 | Assaf S, and Davis LM, 2019 | Women’s modern contraceptive use in sub-Saharan Africa: does men’s involvement matter?. | Wrong setting |
| 180 | Sorsa S et al., 2002 | Health problems of street children and women in Awassa, Southern Ethiopia. | Wrong publication date |
| 181 | Terefe A, and Larson CP, 1993 | Modern contraception use in Ethiopia: does involving husbands make a difference?. | Wrong publication date |
| 182 | Beekle AT, and McCabe C, 2006 | Awareness and determinants of family planning practice in Jimma, Ethiopia. International Nursing Review. 2006 Dec;53(4):269-76. | Wrong publication date |
| 183 | Regassa N, 2007 | Socio-economic correlates of high fertility among low contraceptive communities of southern Ethiopia. | Wrong publication date |
| 184 | Ayalew T et al., 1993 | Assessment of unmet needs and the demand for family planning in Addis Ababa. | Wrong outcomes |
| 185 | Tilahun D et al., 2010 | Predictors of emergency contraceptive use among regular female students at Adama University, Central Ethiopia. | Wrong population |
